# Monte Carlo investigation of dose distribution of uniformly and non‐uniformly loaded standard and notched eye plaques

**DOI:** 10.1002/acm2.14149

**Published:** 2023-09-22

**Authors:** Oleksii Semeniuk, Victor Malkov, Marc J. P. Chamberland, Robert A. Weersink

**Affiliations:** ^1^ Radiation Medicine Program Princess Margaret Cancer Center Toronto Canada; ^2^ Department of Radiation Oncology University of Toronto Toronto Canada; ^3^ Department of Radiation Oncology University of Vermont Medical Center Vermont USA; ^4^ Department of Medical Biophysics University of Toronto Toronto Canada; ^5^ Present address: Department of Radiation Oncology Rhode Island Hospital USA

**Keywords:** eye plaque brachytherapy, Monte Carlo dosimetry, non‐uniform loading, notched eye plaques

## Abstract

To investigate the effect of using non‐uniform loading and notched plaques on dose distribution for eye plaques.

Using EGSnrc Monte Carlo (MC) simulations, we investigate eye plaque dose distributions in water and in an anatomically representative eye phantom. Simulations were performed in accordance with TG43 formalism and compared against full MC simulations which account for inter‐seed and inhomogeneity effects.

For standard plaque configurations, uniformly and non‐uniformly loaded plaque dose distributions in water showed virtually no difference between each other. For standard plaque, the MC calculated dose distribution in planes parallel to the plaque is narrower than the TG43 calculation due to attenuation at the periphery of the plaque by the modulay. MC calculated the dose behind the plaque is fully attenuated. Similar results were found for the notched plaque, with asymmetric attenuation along the plane of the notch. Cumulative dose volume histograms showed significant reductions in the calculated MC doses for both tumor and eye structures, compared to TG43 calculations. The effect was most pronounced for the notch plaque where the MC dose to the optic nerve was greatly attenuated by the modulay surrounding the optic nerve compared to the TG43. Thus, a reduction of optic nerve D95% from 14 to 0.2 Gy was observed, when comparing the TG43 calculation to the MC result. The tumor D95% reduced from 89.2 to 79.95 Gy for TG43 and MC calculations, respectively.

TG43 calculations overestimate the absolute dose and the lateral dose distribution of both standard and notched eye plaques, leading to the dose overestimation for the target and organs at risk. The dose matching along the central axis for the non‐uniformly loaded plaques to that of uniformly loaded ones was found to be sufficient for providing comparable coverage and can be clinically used in eye‐cancer‐busy centers.

## 1 INTRODUCTION

Low dose rate (LDR) brachytherapy using temporarily implanted plaques is currently the most common treatment option for a number of early stage and medium‐sized intraocular conditions, including ocular melanomas, retinoblastoma, choroidal hemangioma, select choroidal metastases, and exudative macular degeneration. LDR uniquely offers equivalent tumor control to enucleation, while preserving the eye and vision function.^1^, ^2^, ^3^, ^4^, ^5^ Most cancer centers treat only low numbers of eye cancer patients per year where these treatments follow Collaborative Ocular Melanoma Study (COMS) guidelines and use a new batch of same‐strength seeds ordered for each case. (See Figure S1 for a cross‐sectional schematic of the plaque geometry).

In high‐throughput centers like ours, treating over 100 patients with choroidal melanomas annually, a procurement of new seeds for every patient proves to be cost inefficient.^6^ Therefore, plaque loadings using seeds of different source strengths has been adopted. Clinically, the non‐uniform ^125^I seed loading is modeled with the Pinnacle (Philips Radiation Oncology Systems, Fitchburg, WI) treatment planning system (TPS) calculated using TG43 methodology to the prescription point located at the tumor apex with prescription heights limited to between 4–10 mm. The planning goal is to match the dose distribution using non‐uniformly loaded plaques to the analogously sized uniformly loaded plaque. While the loading may be non‐uniform, we have found that symmetric loading patterns aid in matching to the uniform dose distribution.

Approximately 10% of our center's eye plaque cases (i.e., ∼10 cases per year) treat tumors close to the optic nerve. In these cases, a combination of notched plaques and non‐uniform source distribution is used to target the tumor while minimizing dose to the optic nerve. To fully cover the tumor with the required dose, the plaque diameter is much larger than the tumor, and the plaque is positioned so that the optic nerve is within the notch. The modulay in the notch is intended to reduce dose to the optic nerve. A further 10% of treatments use a standard plaque that is, asymmetrically loaded to achieve a similar dose distribution. The steep dose gradient and pronounced effect of material heterogeneities (not accounted in Pinnacle TPS) pose significant challenges for accurate eye plaque dosimetry for these cases that is, largely studied for uniformly loaded standard COMS eye plaques.^7^, ^8^, ^9^, ^10^, ^11^, ^12^

While Monte Carlo (MC) dose calculations for eye plaque therapy has been examined in detail for standard COMs plaques, in this work, we investigate the MC dose distributions in water and an eye model phantom for the two planning scenarios within our clinical practice. The first is non‐uniformly loaded standard plaques designed to match the dosimetry of the uniform COMS plaque. The results of this scenario will be used to validate our MC model by comparison with other literature values and to assess the impact of using non‐uniform versus uniform loading, with the hypothesis that the dosimetric impact is negligible. The second scenario is non‐uniformly loaded notched plaque, designed to reduce dose to the optic nerve. Here we compare TG43 and MC dose calculations by examining the impact of dose to tumors and organs‐at‐risk (OARs).

## 2 METHODS AND MATERIALS

### 2.1 Eye plaque design

**FIGURE 1:**
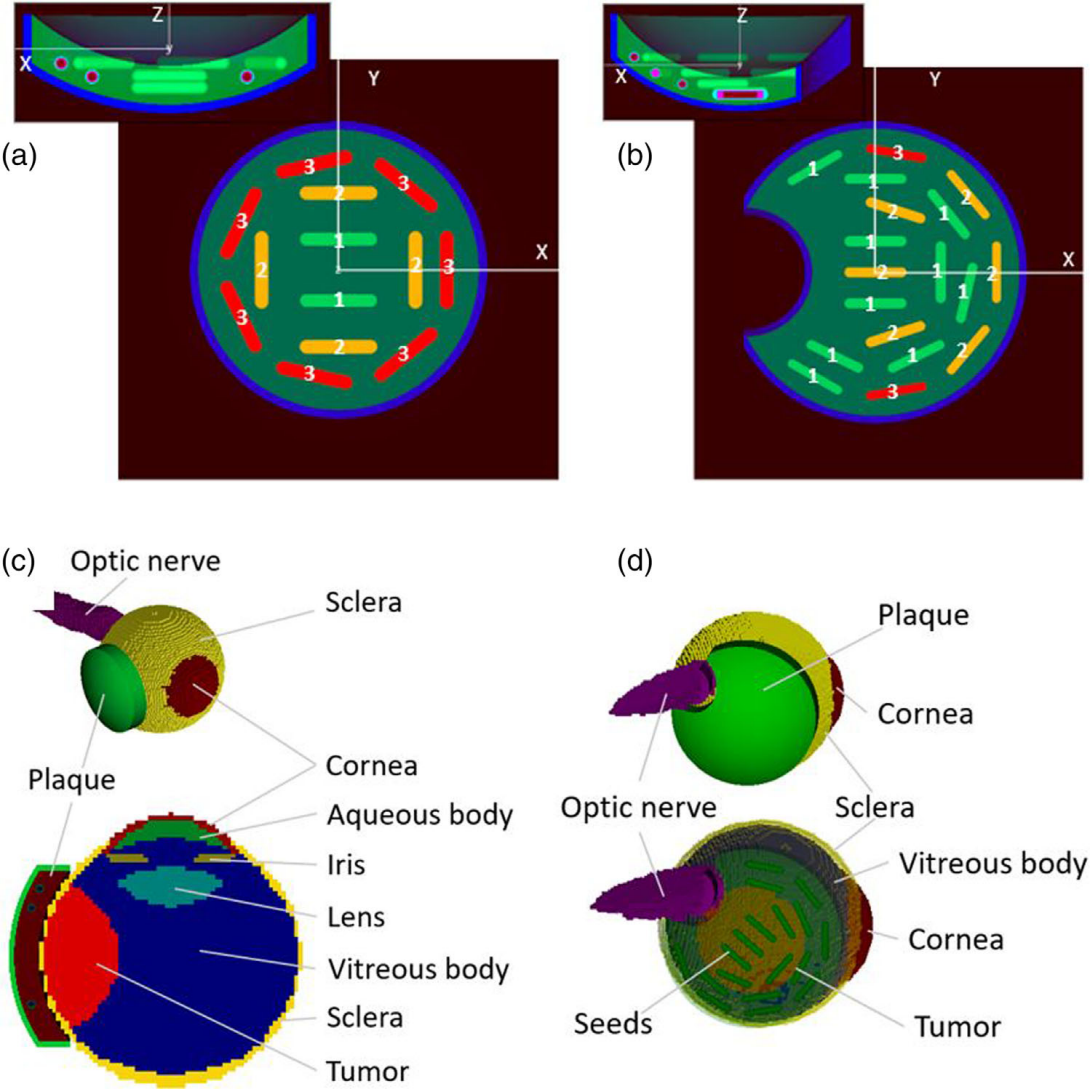
The standard 16 mm COMS (a) and 20 mm notched (b) plaque designs. The inset to the picture shows the cross‐sectional view of the plaque. For the notched plaque in (b), the radius of the notch, *r* = 4.0 mm and offset *a* = 9 mm. (c) MC rendering of the eye model with the laterally located tumor and standard 16 mm COMS plaque; (d) The eye model with the tumor abutting the optic nerve and a 20 mm notched plaque. The source strength are given in Table 1. COMS, Collaborative Ocular Melanoma Study; MC, Monte Carlo.

Two eye plaque designs were simulated in this study, a standard 16 mm diameter COMS plaque and a 20 mm diameter notched plaque. The standard 16 mm COMS plaque geometry (Figure 1a) was simulated with plaque radius, modulay and silastic thicknesses of 12.3, 0.5, and 2.25 mm, respectively. The notched plaque (Figure 1b) was produced by introducing a concave notch to the standard 20 mm COMS plaque. Based on physical measurements of the actual notched plaque, the notch was modeled as a cylindrical cut‐out of the plaque 8 mm in diameter centered at 9 mm from the plaque center along the −x axis. The notch wall thickness was the same as that of modulay.^13^, ^14^

### 2.2 Simulation media

The dose distributions from both plaques were evaluated in a water box and an eye phantom. The plaques were positioned in the center of the (10 cm^3^) water box (i.e., the edge of the plaque along its central axis is at (0; 0; −1 mm)) with 1 mm^3^ voxels, within which the dose was scored. The simulated eye phantom, including the uniformly and non‐uniformly loaded plaques, was modeled with the human CT data from the Cancer Imaging Archive.^15^ The original image was 20 cm^3^ × 20 cm^3^ × 23 cm^3^, with the voxel dimensions of 0.39 mm^3^ × 0.39 mm^3^ × 0.4 mm^3^. The ocular structures were created in the RayStation (RaySearch, Stockholm, Sweden) TPS using defined contouring shapes for each ROI, as described elsewhere.^16^ This was done largely due to limitations of eye geometry definition within the MC. The details of the eye and tumor model are given in Supporting materials. Two tumor locations were modelled in this work. For investigation of dose distribution with standard plaques, the tumor was located on the medial side of the left eye, symmetrically along the *z*‐axis. Alternatively, when investigating the notched plaque design, the tumor was located posteriorly, abutting to the optic nerve.

### 2.3 Treatment planning

The Pinnacle TPS was used to generate the clinical TG43 – based plans using the method outlined in detail elsewhere.^17^ In summary, the planning goal is to match the dose distribution using non‐uniformly loaded plaques to the analogously‐sized uniformly loaded plaque. The comparison between the uniform and non‐uniform dose distributions is made by creating an additional uniformly loaded plan and adding a second reporting point located on the uniform plan at the intersection of the prescription dose line and the inner sclera. Dose at this same location in the clinical plan must be within 5% of the prescription dose.

As previously noted, the first case was a typical standard COMS case. The size of the plaque was selected to extend 2 mm beyond the edge of the tumor as per our clinical protocols, with the prescription point set 5 mm away from the inner sclera along the plaque's central axis along *z*‐direction (i.e., CAX point). Two plans were created in the TPS. One plan was a uniformly loaded plaque matching the standard COMS dose distribution. The second plan used the non‐uniform loading, based on the seed strengths available in our clinic. Besides the prescription point, a second dose calculation point was located at the intersection of the 85 Gy isodose line and the inner sclera, referred to as the “peripheral comparison” point. Both points were used to compare the dose distribution between a theoretical uniform plan and the actual non‐uniformly loaded plan. The distribution of seed strength assignment was manually modified until the dose at both dose measurement points was within 5% of the prescription value, that is, 85 Gy ± 4.25 Gy.

In the second clinical example, a 20 mm notched plaque was chosen because this plaque provided excellent coverage of the 12 mm diameter tumor once it was positioned around the optic nerve. Rather than attempting to mimic a uniform loading (as in the case above), a non‐symmetric loading was deliberately used to provide greater dose coverage over the tumor while minimizing dose to the remainder of the eye. In typical clinical practice, the second dose calculation point is not used for asymmetrically loaded or notched plans, since the plan intention is not expected to correspond to a standard COMS delivery. However, it was included in this study to provide an additional comparison point using the different dose calculation methods.

Table 1 shows the source strengths used for generating the treatment plans with the uniform and non‐uniform loading of standard and notched eye plaques. The dose comparison point for the standard COMS 16 mm and 20 mm notched plaques were (7.7 mm; 0; 3 mm) and (9.5 mm; 0; 5.1 mm), respectively.

**TABLE 1:** Source strengths for standard and notched plaques, modeled in Pinnacle treatment planning system.

Seed location (see Figure 1)	16 mm COMS		20 mm notched	
	Uniform	Non‐uniform	Uniform	Non‐uniform
1	3.15 U	4.4 U	3.56 U	3.46 U
2	3.15 U	2.14 U	3.56 U	1.76 U
3	3.15 U	1.02 U	3.56 U	0.85 U

Abbreviation: COMS, Collaborative Ocular Melanoma Study.

### 2.4 MC simulations

**FIGURE 2:**
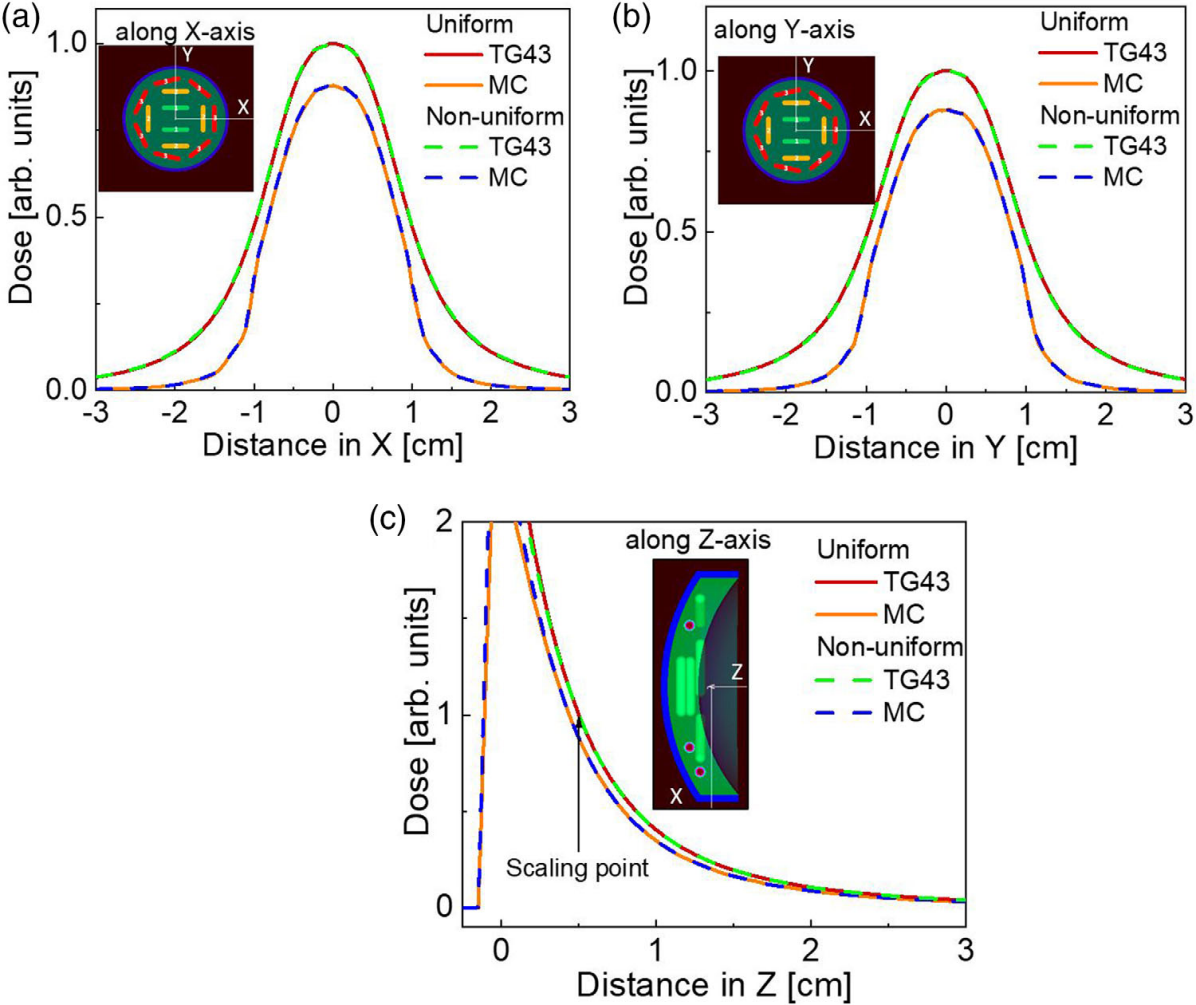
Dose distributions in water for uniformly and non‐uniformly loaded 16 mm COMS eye plaques along *x*—(a), *y*—(b) and *z*—(c) *z* axis of the plaque (see Figure 1a) with the relative dose scaled to TG43 calculation. Cross‐sections are through the prescription point at (*x*,*y*,*z*) = (0,0,5.0) mm. The effect of plaque shielding is seen on (c). Note that the scale in (c) is restricted primarily to show the dose within the eye, and hence doesn't show the dose further along –*z* values. The source strengths are given in Table 1. COMS, Collaborative Ocular Melanoma Study.

**FIGURE 3:**
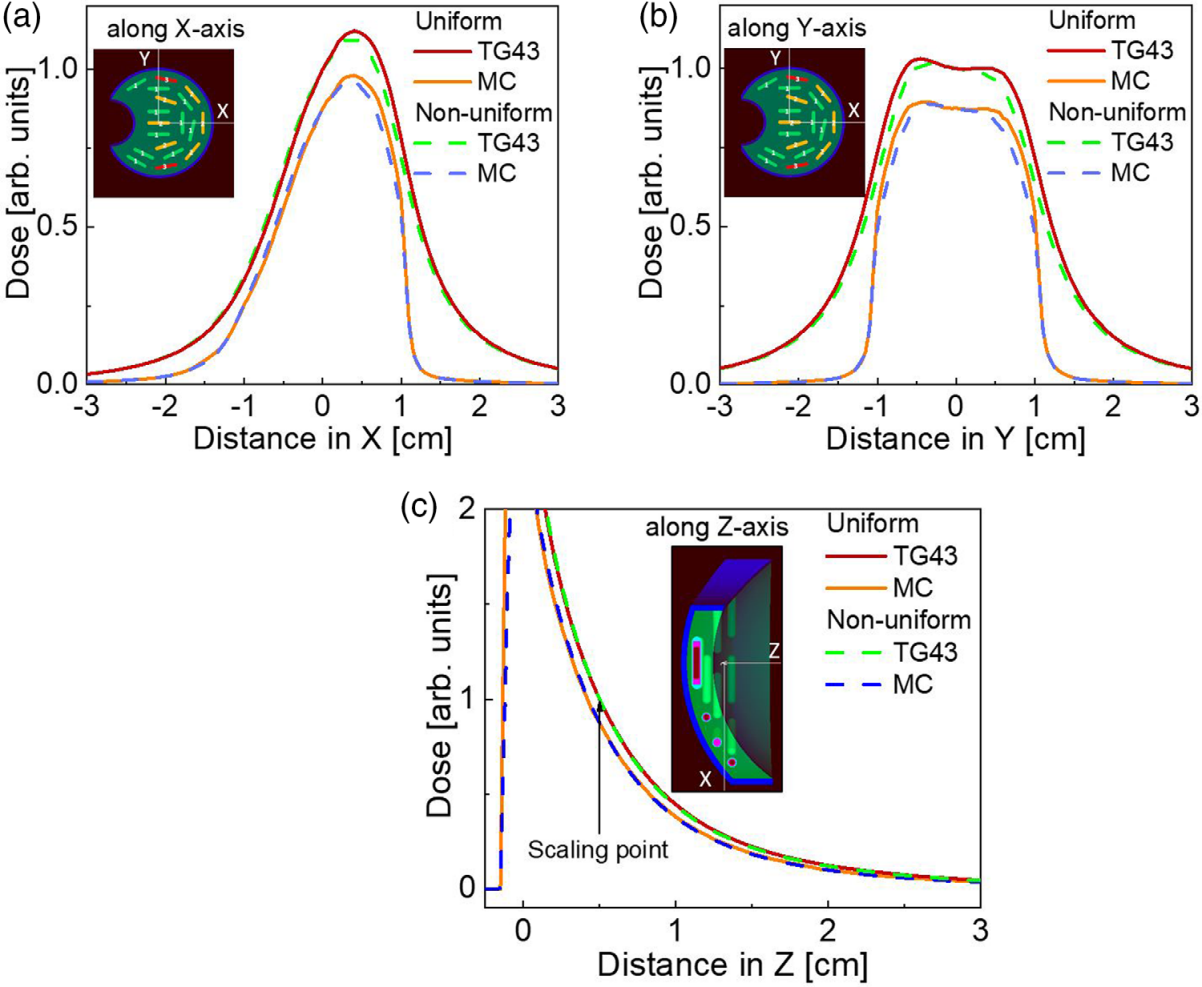
Dose distributions in water for non‐uniformly loaded 20 mm eye plaques along *x*—(a), *y*—(b) and *z*—(c) *z* axis of the plaque (see Figure 1b). The effect of plaque shielding is seen on (c). Note that the scale in (c) is restricted primarily to show the dose within the eye, and hence doesn't show the dose further along –*z* values. The source strengths are given in Table 1. COMS, Collaborative Ocular Melanoma Study.

The calculation of dose profiles for COMS and notched eye plaques in water and in the eye phantom were performed using the egs_brachy package of the EGSnrc MC code.^18^, ^19^ Simulation details are provided in Table 2. MC simulations were performed in water to simulate the standard TG43 dose calculation for comparison against TPS results. Further, MC dose distributions were calculated for the eye model and accounted for inter‐seed and inhomogeneity effects due to tissue and the presence of the plaque. For dose scoring in water, the plaque and simulation media were defined directly in the egs_brachy package, with the electron transport turned off in the phantom. For dose scoring in the patient, the EGSnrc CTCREATE package was used to convert the CT data of the patient with an input phantom file for the egs_brachy simulation.^20^ The default ramp for converting CT values to material and density was edited with custom CT numbers for tumor and ocular structures, with their elemental compositions and densities based on previously published literature.^21^, ^22^, ^24^, ^25^ For the standard plaque simulation, the plaque was positioned against the eye, symmetric to the tumor in both anterior/posterior and superior/inferior directions and along the same central axis (Figure 1c). For the tumor abutting the optic nerve, the notched plaque was positioned centrally to the tumor with the 1 mm gap between the notch and the optic nerve (see Figure 1d). Within the geometric definition of the eye‐plaque in the MC model, the creation of a true empty concavity is limited. Therefore, for simplicity the cut‐out volume of the eye plaque was modeled as an optic nerve cylinder running parallel to the notch external wall, surrounded by 1 mm of the soft tissue. The dose was not scored in the cut‐out region. However, since modulay provides complete attenuation of the ^125^I radiation (see Figures 2c and 3c), this is believed to be insignificant for the MC cumulative dose‐volume histograms (cDVH) calculations.

**TABLE 2:** Parameters of Monte Carlo simulations.

Parameter	Description	Reference
Code	EGSnrc (v2021 – master)	
egs_brachy (v 0.9.1)	[18, 19]	
Validation	validated against dose calculations of all water and full heterogeneity against published data	[7]
timing	∼3 h on 30 Linux cores (simulation time would be improved through additional use of variance reduction techniques, not used here)	
Source	^125^I OncoSeed 6711 (egs_brachy model) with standard and notched COMS plaque.	[18, 19]
simulation parameters	PCUT = 0.001 MeV	
ECUT = 1.5 MeV		
tracklength scoring		
single generator mode		
cross‐sections	XCOM database (AE = 0.512 MeV, AP = 0.001 MeV)	
Mass‐energy absorption coefficients calculated using the EGSnrc application ‘g’		
histories (statistical uncertainty with parameter *k* = 1)^18^	1e10 histories (<1% within all dose scoring regions)	

Finally, the source strengths were extracted from the Pinnnacle TPS, as given in Table 1. The associated relative strengths were used in MC calculations. To expedite the calculation time, the phantom size was trimmed to 6 cm^3^ × 6 cm^3^ × 5 cm^3^ in size, while original voxels dimensions were preserved. The phantom was placed within a (10 cm^3^) water box to provide adequate scattering conditions, however the dose was scored only in those voxels corresponding to the phantom.

## 3 RESULTS

### 3.1 Dose distribution in water

The doses at the comparison point from Pinnacle TPS and MC calculations for non‐uniformly loaded standard and notched eye plaques are given in Table 3. The TG43‐based MC calculations in water were in good agreement with those computed by the Pinnacle TPS. This is expected, since Pinnacle calculates the dose distribution in the water‐equivalent medium using TG43 method.

**TABLE 3:** Computed doses for non‐uniformly loaded plaques in water at the peripheral comparison point. The dose values are given as a percentage of the dose at CAX prescription point in TG43 calculations.

Plaque	Pinnacle TPS [%]	MC TG43 calculation [%]	MC calculation [%]
16 mm COMS	100.6	100.9	84.3
20 mm notched	92.9	92.3	73.4

Abbreviations: COMS, Collaborative Ocular Melanoma Study; MC, Monte Carlo; TPS, treatment planning system.

The dose distributions in water for uniformly and non‐uniformly loaded standard plaques are shown in Figures 2a–c. The dose distributions were scaled to the dose at the CAX prescription point, lying on the central axis of the plaque, 5 mm away from its center. The dose profiles along *x*‐ and *y*‐ axes shown in Figure 2a and b, respectively, were taken at orthogonal planes crossing the prescription point. As seen from Figure 2, the TG43 calculations overestimated the distribution for both uniformly and non‐uniformly loaded plaques. Specifically, our data showed that TG43 calculation overestimates the dose by 12.3% and 14.2% at 5 mm and 1 cm from the plaques center, respectively, which is in good agreement with previous calculations.^7^ Previous studies have shown this is predominantly due to attenuation by the silastic holding the seeds in place.^9^, ^11^, ^23^ The most notable difference with the MC dose is the pronounced effect of plaque attenuation, especially along the negative *z*‐axis. As seen from Figure 2c, the dose behind the plaque there is practically complete attenuation of ^125^I maximum photon energy. In contrast, along the positive *z*‐direction (towards the eye) discrepancies are minimal after first 2 cm from the silastic, where TG43 methods provide higher dose. The relative dose calculated using MC generally follows the TG43 calculation, similar to previous studies.^7^, ^16^ The relative dose distribution along the *x*‐ and *y*‐axes is also narrower for the MC calculations beyond the radius of the plaque. For both TG43 and MC calculations, the non‐uniformly loaded plaque dose distributions closely resemble those with uniform loading.

The dose distributions with the notched plaques in water are shown in Figures 3a–c. The presence of the notch shifts the location of maximum dose away from the center of the plaque along the positive *x*‐axis (Figure 3a), that is, away from the notch. The dose distribution is also seen to be marginally perturbed along the *y*‐axis due to the non‐uniform seed placement within the plaque (Figure 3b). The effect of plaque attenuation, observed along the center axis of the plaque (Figure 3c) is similar to that observed with the standard COMS plaque, exhibiting essentially complete shielding by the modulay.

### 3.2 Dose‐distribution in patient

**FIGURE 4:**
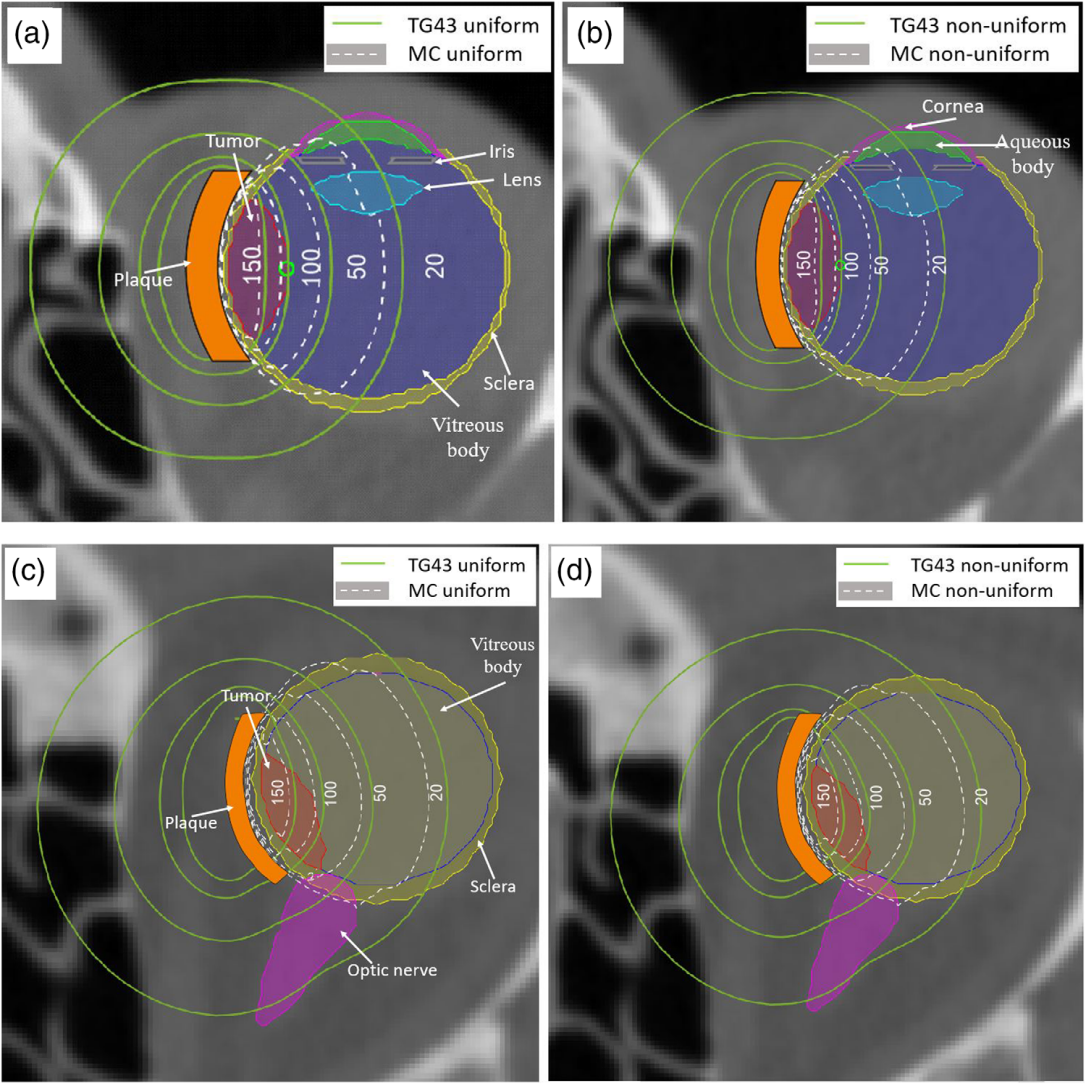
Dose distribution in the patient with (a) uniformly loaded standard 16 mm; (b) non‐uniformly loaded 16 mm; (c) uniformly loaded notched 20 mm; (d) non‐uniformly loaded notched 20 mm plaques in TG43 and MC calculations. The central slice of the eye was selected for visualization. The normalization point, lying 5 mm away from the plaque center (coinciding with tumor apex), corresponds to 85 Gy (100% iso dose line) in the TG43 simulation is shown with green circle. The slice containing the optic nerve was selected for better visualization of sparing effect of the notch. MC, Monte Carlo.

The effect of loading pattern on dose distribution for standard and notched plaques is shown in Figure 4. The doses were scaled to provide 85 Gy at the prescription point for the TG43 calculation.^16^ As seen from Figure 4, TG43 calculations overestimate the dose distribution in the patient for both types of plaques with TG43 isodose lines symmetrically extending further in the patient than observed for MC calculations. The isodose lines of MC simulations, both for uniform and non‐uniform loading, are seen to be confined within the sclera by the presence of the modulay. For the notched plaque, modulay at the notch strongly limits high doses of radiation to the optic nerve, although lower radiation doses are still observed at the anterior portion of the optic nerve.

**FIGURE 5:**
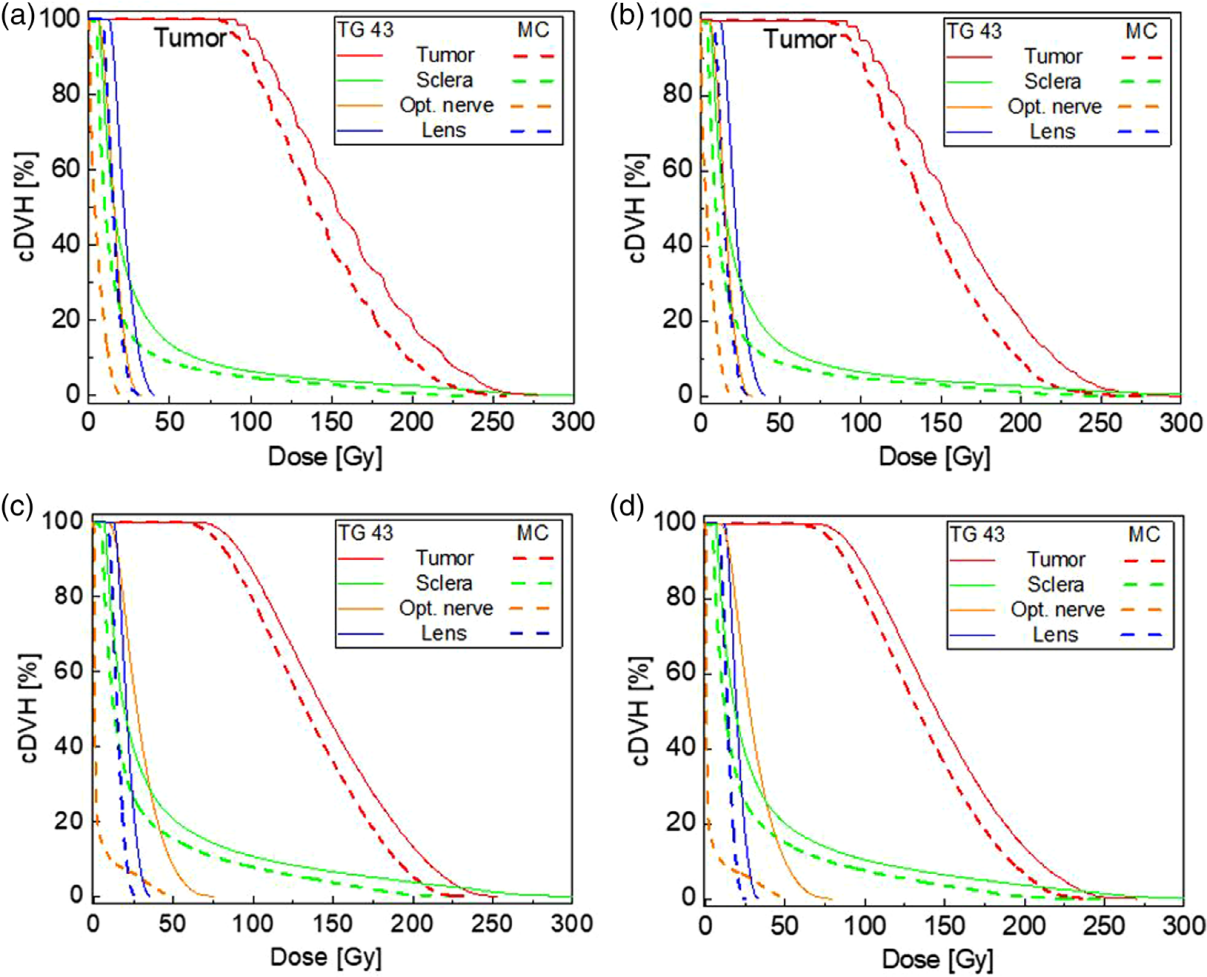
cDVH curves in the patient with (a) uniformly loaded standard 16 mm; (b) non‐uniformly loaded 16 mm; (c) uniformly loaded notched 20 mm; (d) non‐uniformly loaded notched 20 mm plaques. cDVH, cumulative dose‐volume histograms.

Figures 5a,b show the cDVH curves for the uniformly and non‐uniformly loaded standard 16 mm COMS plaque, respectively. The curves for non‐uniform loading essentially overlay those for uniform loading with a pronounced difference between TG43 and MC calculations. The TG43 calculations overestimate those using MC, which is in a good agreement with the data published by Lesperance and colleagues.^16^ Overall, the cDVHs for the standard plaque are qualitatively similar, with the TG43 calculated doses higher by ∼13% than those obtained with MC.

Figures 5c,d shows the cDHV results for the notched plaques. Overall, the trend with dose overestimation with TG43 calculations is similar to that observed with the standard plaques. The major qualitative difference lies in the optic nerve sparing. This organ lies very close laterally to the plaque, while the rest of the ocular structures are located near the plaque opening. The notch spares the optic nerve, leading to lower deposited doses than calculated by TG43, since the latter does not account for modulay attenuation. The same difference in the MC and TG43 calculations is observed with both uniform and non‐uniformly loaded notched plaques. Table 4 provides further insight into exposure of the tumor and OARs with both types of plaques.

**TABLE 4:** The tumor coverage and organ sparing with 16 mm standard and 20 mm notched non‐uniformly loaded plaques.

	16 mm COMS plaque							
	Tumor		Lens		Sclera		Optic nerve	
	TG43	MC	TG43	MC	TG43	MC	TG43	MC
D1% [Gy]	263.4	239.5	38.5	27.4	260.3	196.7	28.7	17.1
D95% [Gy]	99.4	93	14.2	9.7	7.1	4.8	8	0.5

	20 mm notched plaque							
D1% [Gy]	243.8	215.2	31.5	22.1	257.1	184.4	67.1	43.2
D95% [Gy]	89.2	77.7	13.8	9.3	7.7	5	14	0.2

Abbreviations: COMS, Collaborative Ocular Melanoma Study; MC, Monte Carlo.

## 4 DISCUSSION

This study used MC simulations to investigate dose distributions in standard COMS and notched plaques. There were two aims in performing the calculations. The first was to compare non‐uniformly and uniformly loaded plaques, since our institution uses non‐uniformly loaded plaques, rather than the standard uniformly‐loaded COMs plaques, to achieve the same dosimetric intent. The second was to investigate the dose impact of the plaque shape on the dose distribution, in particular the use of notched plaque placed around the optic nerve. Simulations were performed for dose in water, analogous to TG43 calculations and in a realistic patient scenario accounting for all material properties. As expected, the MC simulated doses in water were lower than those predicted by the TG43 calculations. This trend has been shown by Lesperance et al. for uniformly loaded standard plaques and now confirmed for the non‐uniformly loaded plaques (Figure 4). Indeed, for uniformly loaded 16 mm COMS plaque, the D20% for tumor and lens were reported to be overestimated by TG43 calculations by ∼5.1% and ∼47.7%, respectively. We used similar (although not identical) eye model and received D20% overestimation of ∼11.7% and ∼45.2% for the same structures.^16^ The dosimetric differences are associated with the selection of different voxel size ((0.5 cm) 3 used by Lesperance and colleagues vs. 0.39 cm × 0.39 cm × 0.4 cm in this study) and corresponding volume averaging effects. It should be noted that this is a rather simplistic scenario of laterally‐located tumor treated with generic COMS plaque, that produces circular symmetric dose distribution. As such, the observed differences between TG43 and MC are largely associated with the differential attenuation of various oracular structures and silastic. The effect of inter‐seed and plaque wall attenuation exerts little effect on dose distribution in the eye. Thus, for the standard plaque, the impact of dose to the eye and OARs is primarily an issue of scaling, such that the shape of the cDVH curve calculated using TG43 is similar to the MC cDVH curve, with the TG43 calculation overestimating doses by approximately 10%−20% compared the MC results.

With respect to uniform versus non‐uniform loading patterns for standard plaques, as seen from Figures 2 and 5, they provide nearly identical dosimetric effect for the standard plaques. A good agreement was observed between comparison point doses (see Table 3), confirming adequate dose modeling with Pinnacle TPS for the water‐equivalent cases. This expected result validates our clinical practice of using non‐uniform loading for standard COMS delivery since they mimic the dose distribution of the uniform loading.

MC calculations of the notched plaque highlights substantial differences compared to the TG43 computations (Figure 3 and 5c,d), particularly for the optic nerve. While TG43 still overestimates the dose, the lack of cylindrical symmetry means that the differences between TG43 and MC dose is not simply a scaling factor along the central axis of the plaque. Instead, significant sparing of the optic nerve is observed due to the almost complete shielding provided by the modulay. A comparison of the optic nerve D1% shows a large reduction in volume treated at higher doses, which is clinically relevant as this is related to permanent and irreversible blindness.^1^ Further, there is a reduction of optic nerve volume irradiated, demonstrated by a reduction of D20% from 41.7 to 3 Gy and D95% from 14 to 0.2 Gy when comparing the TG43 calculation to the MC result. The presence of the modulay surrounding the optic nerve provides exceptional radiation protection to this OAR and may allow for more aggressive treatment planning of tumors using notched plaques near the optic nerve. Tumor D95% using TG43 is under 90 Gy and it can be seen in Figure 5 that the TG43 dose calculation does not fully cover the tumor in the plan, even though the dose is still 85 Gy at the prescription point. Placing the notch tighter against the optic nerve could correct this underdosing, but small placement errors could also lead to large variations in dose coverage given the large dose gradients at the notch. Full 3D imaging of the eye may be necessary for these cases, along with further evaluation of acceptable planning target volumes. Further modelling is needed to assess the impact of such an approximation on the dosimetry of the tumor and OARs.

Overall, our results generally indicate that when achieving the same prescription target coverage as in MC calculation, the TG43 methods overestimate the OAR doses. This is particularly notable for the case of the optic nerve and notched eye plaque and may provide the opportunity for further dose optimization when taking into account the detailed MC dose calculation. The extent of optimization would be patient specific and motivates the integration of MC‐based dosimetry into clinical eye plaque treatment planning. Such a process can be integrated into the patient workflow by contouring ocular structures on patient CT or MRI data. While the calculations shown here are patient specific, the dose calculation can be accelerated by assuming all tissue has one attenuation value and using a set of precalculated library seed dose distributions for each source of each plaque and then summing these distributions using available source strengths to match the required dosimetry. It should be noted that current simulations were not optimized for computation efficiency and has rather high uncertainty threshold of <1% in both target and OARs. However, for clinical utilization, a lower uncertainty threshold might be sufficient. In addition, variance reduction/particle recycling techniques allow to reduce computation time in several folds.^18^ The aforementioned methods would allow for more accurate patient‐specific dose calculations, without the extended computation times associated with MC simulations.

## 5 CONCLUSIONS

TG43 calculations overestimate the absolute dose to the prescription point and the lateral dose distribution of both standard and notched eye plaques, leading to the dose overestimation for the tumor and organs at risk (optic nerve, iris, lens, etc.). The dose distributions for the non‐uniformly loaded plaques were an excellent match to dose distributions of uniformly loaded ones and can be clinically used in centers with heavy eye‐cancer patient load. Our MC models of standard and notched plaques models will be further evaluated for treatment planning of patient‐specific eye lesions. A specific future application is to use asymmetrically loaded and notched plaques positioned close to the optic nerve, where MC models will provide more accurate dosimetry to this sensitive structure compared to TG43 methods.

## AUTHOR CONTRIBUTIONS

Robert A. Weersink and Victor Malkov designed the project. Robert A. Weersink provided mentorship and resources needed for the project. Oleksii Semeniuk developed the models, analyzed the data, and wrote the manuscript. Marc J.P. Chamberland made a significant contribution to troubleshooting the simulation and Victor Malkov ran the calculations. All the authors contributed to the conception, data analysis, writing, and revisioning of the manuscript.

## CONFLICT OF INTEREST STATEMENT

The authors declare that there is no conflict of interest.

## Supporting information

Supporting Information

Supporting Information
