# Supplementary figures and images for: Monte Carlo investigation of dose distribution of uniformly and non‐uniformly loaded standard and notched eye plaques
Source: J Appl Clin Med Phys. 2023 Sep 22;24(12):e14149. doi: 10.1002/acm2.14149 (PMC10691642; doi:10.1002/acm2.14149)

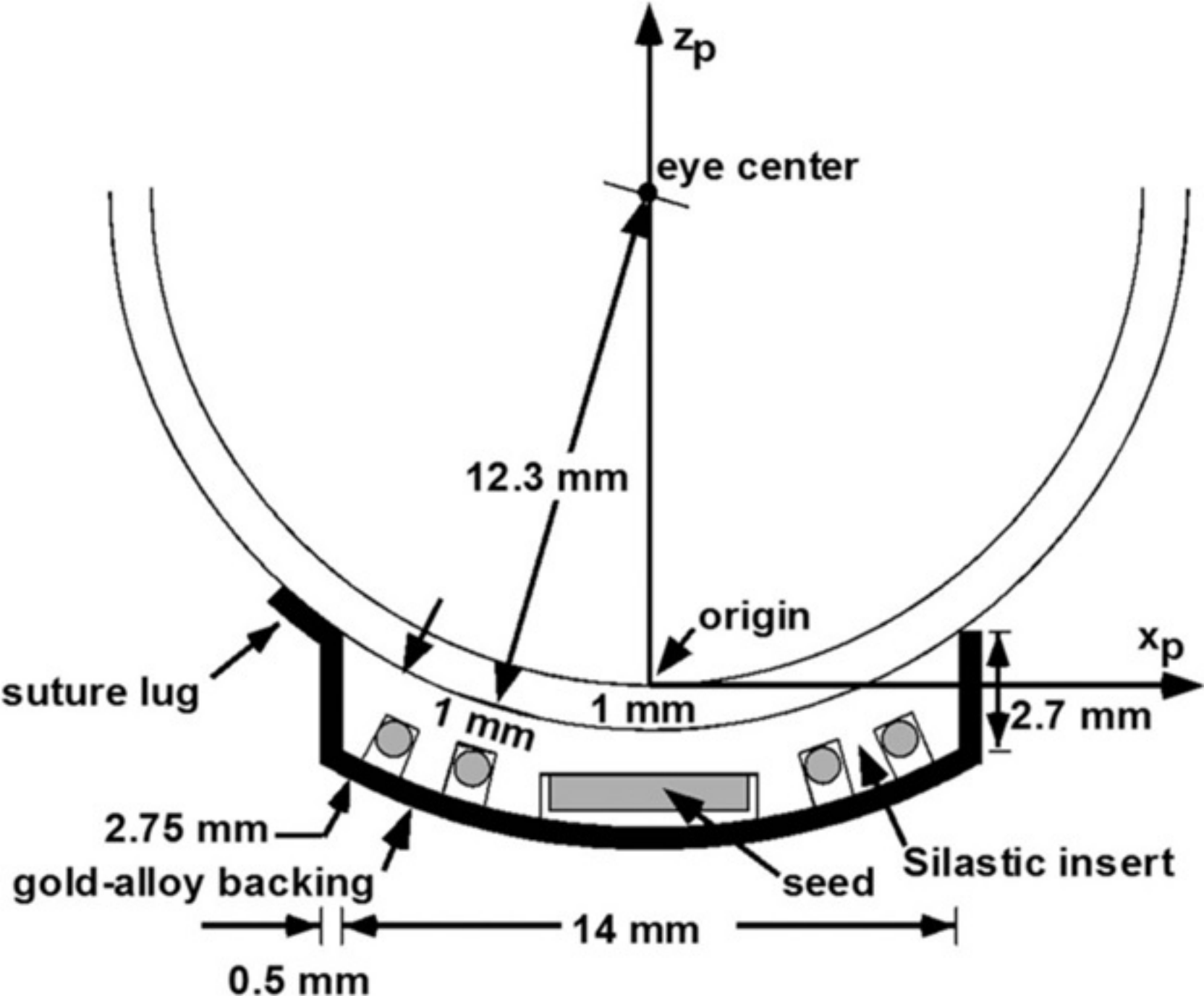

Supplement: Supplementary file 1 — Supporting Information [file ACM2-24-e14149-s001.pdf]
