# Supplementary material for: Monte Carlo investigation of dose distribution of uniformly and non‐uniformly loaded standard and notched eye plaques
Source: J Appl Clin Med Phys. 2023 Sep 22;24(12):e14149. doi: 10.1002/acm2.14149 (PMC10691642; doi:10.1002/acm2.14149)
Supplement: Supplementary file 2 — Supporting Information [file ACM2-24-e14149-s002.pdf]

Monte Carlo Investigation of dose distribution of uniformly and non-uniformly loaded standard and notched eye plaques

Supporting Materials

In our eye model, the outer sclera (i.e. the size of the eye) was chosen to be 24 mm in diameter, which represents the median value of the adult eye size, ranging from 21 to 26 mm. The inner sclera (i.e. the outer boundary of the vitreous body) was defined as a union of three spheres 11 mm, 10.7 mm and 10.7 cm in diameter, positioned at the (0; 0; 0), (-1.2 mm; 0; 0) and (1.2 mm; 0; 0), respectively from the center of the sclera. This allowed for gradual transition of the sclera thickness between 0.3 mm laterally to 1 mm in anterior-posterior directions. The outer cornea was bounded by the two spheres with diameters of 15.6 mm and 13 mm, respectively, shifted by 5.5 mm and 6.3 mm anteriorly, resulting in the cornea thickness of 0.52 mm along the x-axis. The aqueous body of the eye was contoured as a region, sandwiched between the inner cornea sphere and the vitreous body.[22] Posteriorly to the aqueous body lies the iris defined as a 0.6 mm thick disk, bounded by two cylinders 5 mm and 11.7 mm in diameter.[24] The lens is defined as an intersection of two spheres, 20 mm and 12 mm in diameter, centered at (0; 0; -2 mm) and (0; 0; 10.6 mm) in the x direction, providing the lens diameter of 9.5 mm at its widest point. The optic nerve has a cross-section of ~3.5 mm outside of the eye, shrinking to 1.5 mm in diameter at the intersection with vitreous body.[22] Its spatial location and orientation were contoured using CT data. Other ocular structures, such as the fovea and retina, are not included in the eye model since their size is smaller than the phantom resolution. The tumor was modelled after the most common dome-shaped choroidal melanoma.[25] It was defined as the intersection of inner sclera and a sphere 7.3 mm in diameter, such that at its widest point the tumor diameter was 12 mm and its apex lies 5 mm inward from the inner sclera.

Figure S1: Cross-sectional schematic of COMs eye plaque, showing the gold-alloy backing (modulay) and Silastic insert with seed grooves accommodating seeds (gray circles and rectangle) in idealized positions. The Silastic insert is 1 mm thick between the groove and the concave surface of the insert. The coordinate system origin is located at the inner sclera along the plaque central axis. The z_p-axis is pointing toward the eye center, the x_p-axis points away from the suture lug (eyelets), and the y_p axis (not shown) points into the figure. (From Ref. 4, with permission.)
